# Health Risks, Fatigue, and Loyalty in Food Delivery Apps: The Moderating Power of Nutrition Disclosure and Chatbots

**DOI:** 10.3390/foods14183253

**Published:** 2025-09-19

**Authors:** Joonho Moon, Yunho Ji

**Affiliations:** Department of Tourism Administration, Kangwon National University, Chuncheon 24341, Republic of Korea; joonhomoon@kangwon.ac.kr

**Keywords:** health risk, fatigue, loyalty, nutrition, disclosure, chatbot, food delivery app

## Abstract

The healthiness of food can become a weakness of food delivery app services. Hence, this research aims to examine the relationships among health risk, fatigue, loyalty, nutrition disclosure, and chatbot information in the context of food delivery apps. In particular, this study investigates the moderating roles of nutrition disclosure and chatbot information on the effect of health risk on fatigue, using information overload as the theoretical underpinning. Data were collected through an online survey on the Clickworker platform, with 364 valid responses from consumers in the U.S. market. This study employed Hayes’s PROCESS Macro Model 7 to test the research hypotheses. The results indicate that loyalty is significantly affected by both health risk and fatigue. Furthermore, the findings demonstrate significant moderating effects of nutrition disclosure and chatbot information on the relationship between health risk and fatigue. This study contributes to the literature by elucidating the associations among the five constructs and additionally provides important managerial implications.

## 1. Introduction

Researchers have emphasized that a key limitation of food delivery applications is their limited provision of healthy food options [1,2,3]. Accordingly, it is important for platform managers to develop strategies that mitigate the predominance of less healthy offerings. Building on this premise, the present research conceptualizes food-related health risk as the independent variable, fatigue as the mediating variable, and loyalty as the dependent variable, thereby providing a comprehensive understanding of consumer behavior in food delivery app usage.

While technological advancements have enhanced convenience, they have simultaneously increased users’ mental fatigue due to the need to process larger volumes of information and adapt to digital devices [4,5]. Examining mental exhaustion in the context of digital food platforms thus offers valuable insights. Although Ahn [6] presented findings on user fatigue in food delivery apps, more detailed investigations in this area have remained relatively scarce. Loyalty is adopted as the dependent variable given its strong association with firm revenue and its frequent use as a primary outcome in management research [7,8,9]. To explore the relationship between loyalty and fatigue, this research employed cognitive load theory. Cognitive load theory claims that when individuals expend substantial mental energy in decision-making, negative behaviors may result [10,11]. This provided a basis for inferring the potential for negative consumer behaviors associated with fatigue.

Next, this research investigates the moderating roles of nutritional information disclosure and chatbot-provided information. Although digital platforms offer diverse menu information, they are often criticized for promoting unhealthy food. Disclosure of nutritional information may induce hesitation in decision-making, thereby increasing user fatigue. Similarly, chatbots provide supplementary details beyond standard platform information [12,13]. While providing additional information can enhance consumer knowledge, it may simultaneously increase fatigue among users already concerned about food-related health risks. The empirical investigation focused on U.S. consumers, as the U.S. digital food platform market constitutes a substantial economic sector, with a market volume of USD 52.67 billion in 2024 [14]. The market is also highly competitive: Uber Eats expanded its share to approximately 25% by acquiring Postmates and intensifying marketing efforts to attract consumers amid competition from DoorDash, Amazon.com, and Walmart [15]. The combination of a large market and intense competition makes the U.S. context particularly suitable for examining consumer behavior in digital food platforms.

In summary, this research explores the relationships among health risk, fatigue, and loyalty in the context of food delivery app usage, as well as the moderating effects of nutritional information disclosure and chatbot information on the association between health risk and fatigue. Theoretically, it contributes to understanding consumer mental exhaustion and decision-making in digital food services. Practically, the findings offer actionable insights for managers seeking to develop more sustainable and consumer-friendly platforms.

## 2. Review of Literature and Hypotheses Development

### 2.1. Loyalty

Loyalty refers to consumers’ intention to continue engaging with a specific product or service [8,9]. As sustained usage directly contributes to revenue growth, loyalty has become a central focus in management and consumer research [8,16]. In the food sector, prior studies consistently adopted loyalty as a dependent variable. For example, Izquierdo-Yusta et al. [17] examined its determinants among food consumers, while Lee and Han [18] and Pal et al. [7] focused on digital food platform users. Researchers extended the analysis to related contexts, such as online marketing in fresh food [19] and green marketing in fast food [20], using loyalty as a dependent variable. Collectively, this body of work highlights loyalty as a widely employed outcome variable that is central to understanding both food delivery app usage and broader food consumption behavior.

### 2.2. Fatigue and Cognitive Load Theory

Fatigue is commonly conceptualized as a reduction in attentional capacity resulting from the depletion of mental energy [21,22,23]. The existing literature has investigated fatigue across various domains, emphasizing its implications for cognitive performance and decision-making. For instance, Mullette-Gillman et al. [24] demonstrated that fatigue can lead to detrimental shifts in individual decision outcomes. Kok [25] and Polman and Vohs [26] further highlighted that fatigue is often associated with the burden of making multiple choices, which arises in the process of integrating and evaluating information. Likewise, Jia et al. [27] found that individuals experiencing fatigue tend to perceive themselves as being in a suboptimal state, which prompts more cautious decision-making behaviors and, in turn, influences the decision-making process. Prior research additionally applied cognitive load theory to explain fatigue in decision-making and related consumer behavior, arguing that fatigue arose from individuals’ limited cognitive resources [10,11]. Carroll et al. [28] found that, among consumers making food purchases, cognitive overload induced fatigue, which in turn affected individual decision-making. Zimmerman and Shimoga [11] also demonstrated that excessive information provided through food-related advertising depleted consumers’ cognitive energy, leading to fatigue that negatively influenced decision-making and evaluations. These findings indicated that fatigue not only constrains information processing but also plays a critical role in shaping individual decision-making. Additionally, Zheng and Ling [29] argued that information processing through digital platforms induced individual fatigue. In the context of food delivery apps, Ahn [6] documented that usage-related fatigue could influence negative behaviors such as consumers’ switching intentions. However, research focusing on user fatigue in the domain of food delivery apps has remained limited. Building on this theoretical foundation, the present research conceptualizes fatigue as a mediating variable to explain the mechanism through which food-related health risks influence loyalty.

### 2.3. Health Risk

Food exerts a profound influence on individual health, and the consumption of food containing unfavorable nutritional components may pose significant risks [30,31,32]. Prior works argued that such risks explain the increasing consumer preference for healthier food options [32,33,34]. In this regard, health risk in the food domain is directly associated with the consumption of unhealthy items, and scholars have identified obesity as a major drawback of food delivery applications [34,35,36]. Indeed, numerous studies have reported that food delivery platforms primarily promote and sell less healthy food, thereby shaping consumer perceptions of heightened health risk [1,34,37]. Building on this foundation, the present research aims to investigate how perceptions of health risks associated with food delivery applications influence consumer behavior.

### 2.4. Hypotheses Development

Building on prior findings, this study proposes a conceptual model where health risk influences both fatigue and loyalty, with fatigue acting as a mediator and information sources, including nutrition disclosure and chatbot information, as moderators.

Scholars have argued that consumers experience heightened fatigue as a consequence of anxiety associated with risk [38,39]. Wang et al. [39] demonstrated that individuals’ perceived health risks during the COVID-19 pandemic significantly increased fatigue. Bright and Logan [40] likewise found a significant association between risk and fatigue in consumer decision-making. Similarly, You et al. [3] observed that consumers experience greater fatigue in the context of online food purchases as they attempt to mitigate potential risks. In the domain of food services, Shafieizadeh et al. [41] showed that consumers’ mental energy is depleted during information-processing activities, resulting in heightened fatigue. Furthermore, Kuttschreuter et al. [42] highlighted that consumers exert greater cognitive effort in the information search process when they perceive risks associated with food. Accordingly, this study proposes the following hypothesis:

**Hypothesis** **1.** *Health risk positively affects fatigue in the domain of food delivery app usage.*

Building on prior research, it has also been shown that consumers’ anxiety regarding food negatively influences their perceptions of brand loyalty [43]. García-Salirrosas et al. [44] empirically confirmed that consumer loyalty can be strengthened through foods that promote health. Likewise, Osaili et al. [1] and Sun and Moon [2] demonstrated that healthy food choices significantly contribute to consumer loyalty in the context of food delivery app usage. Based on these insights, this study proposes the following hypothesis:

**Hypothesis** **2.** *Health risk negatively affects loyalty in the domain of food delivery app usage.*

Prior research has suggested that individuals experience fatigue from the use of media and other digital devices, which in turn leads to negative consumer behaviors [4,5]. Ji and Jan [45] empirically demonstrated that media-induced fatigue significantly reduced consumer loyalty. Similarly, Zarantonello et al. [36] and Donnan et al. [46] alleged that mental fatigue plays a key role in driving negative decision-making among consumers. Alfina et al. [47] further emphasized that, because consumer fatigue can have adverse effects in marketing contexts, identifying strategies to mitigate these effects is crucial. Thus, this research proposes the following hypothesis:

**Hypothesis** **3.** *Fatigue negatively affects loyalty in the domain of food delivery app usage.*

Previous research defined nutrition information disclosure as the provision of information to assist consumers in making informed choices, such as details about ingredients and calorie content [48,49]. Studies have argued that providing nutrition information enables consumers to obtain more comprehensive knowledge, which in turn facilitates beneficial decision-making [49,50,51]. Moreover, prior research documented that consumers utilize chatbots to acquire necessary information and support their decision-making processes [52,53]. Chatbots are also employed in commercial contexts to provide information tailored to individual needs or to resolve consumer inconveniences [53,54].

The provision of additional information related to the notion of information overload—whether nutritional or transactional—can impose a cognitive burden on consumers, which has been identified as a potential drawback [55,56]. Information overload is defined as a condition in which characteristics of information—such as its volume, complexity, or rate of presentation—exceed an individual’s capacity to process it effectively [56,57]. Scholars claimed that information obtained through nutrition disclosure [50,58] and chatbot consultations [12,13] can contribute to information overload, as it increases the amount of information individuals must process. Scholars further suggested that such excessive information is likely to reduce consumers’ ability to make effective decisions [57,59]. Building on these insights, it can be inferred that the use of nutrition information disclosure and chatbots may increase cognitive demands on consumers, potentially heightening fatigue. Accordingly, the present study proposes the following hypotheses:

**Hypothesis** **4.** *Nutrition disclosure significantly moderates the relationship between health risk and fatigue in the domain of food delivery app usage.*

**Hypothesis** **5.** *Chatbot information significantly moderates the relationship between health risk and fatigue in the domain of food delivery app usage.*

## 3. Method

### 3.1. Research Model

**Figure 1:**
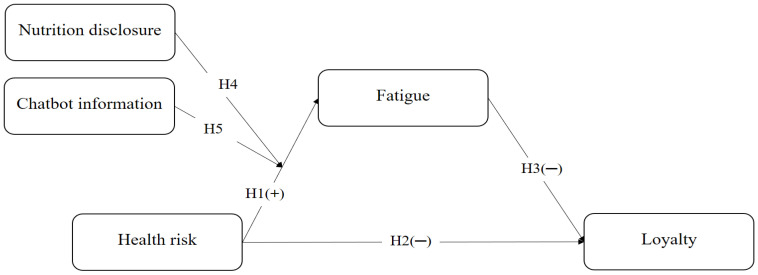
Research model.

Figure 1 illustrates the proposed research model. In this model, health risk is specified as the independent variable, while fatigue is conceptualized as the mediating variable. Loyalty serves as the dependent variable. Nutrition disclosure and chatbot information are introduced as moderating variables that influence the relationship between health risk and fatigue. The model posits that health risk positively affects fatigue, whereas loyalty is negatively influenced by both health risk and fatigue.

### 3.2. Data Collection

Data for this study were collected through an online survey administered via Google Forms. The online survey method was chosen for its flexibility in terms of time and location, which facilitates broader participation. Respondents were recruited through the Clickworker platform (http://clickworker.com, accessed on 26 August 2025), a widely recognized tool for survey-based research [60,61]. The study focused on the United States, given the prominence and active use of food delivery applications in this market. Data collection was carried out between 26 August and 29 August 2025, resulting in 364 valid responses. This sample size is considered adequate for robust statistical analysis, consistent with the rule of thumb that recommends a minimum of ten observations per survey item to ensure reliable inference [62].

Table 1 presents the demographic profile of the survey participants. The proportion of female respondents was 70.6%. In terms of age, the largest group consisted of respondents in their forties (137 individuals, 37.6%). Regarding monthly household income, approximately 59.6% reported earnings below USD 5000. In terms of education, 43.1% of respondents had not completed a college degree, representing the largest subgroup. Finally, 166 respondents indicated that they used food delivery applications less than once per week.

### 3.3. Measurement Items and Data Analysis

Table 2 presents the measurement items employed in this study. All items were assessed using a five-point Likert scale, ranging from 1 = *strongly disagree* to 5 = *strongly agree*. The measurement of loyalty [8,9] and nutrition disclosure [63,64,65] was adapted from prior research and refined in accordance with the research objectives. To develop reliable and context-appropriate items for health risk, fatigue, and chatbot information, two rounds of one-hour consultations were conducted with experts in consumer behavior. Each construct was measured using four items. To minimize potential confusion among respondents, all items were positively worded. The measurement focused specifically on participants’ perceptions, which were based on commonly held stereotypes regarding food delivery services.

The operational definitions of the variables are as follows:

**Health Risk:** The perceived extent to which foods available through food delivery applications are detrimental to health.

**Fatigue:** The degree of mental fatigue experienced by consumers when using food delivery applications.

**Loyalty:** The extent of consumers’ intention to continue using food delivery applications.

**Nutrition Disclosure:** The perceived transparency and usefulness of nutritional information provided by food delivery applications.

**Chatbot Information:** The extent to which consumers rely on chatbots within food delivery applications to obtain information.

This study employed several statistical techniques to analyze the data. First, frequency analysis was conducted to examine the demographic characteristics of survey participants. Next, exploratory factor analysis with varimax rotation was performed, applying a factor loading cutoff of 0.5. Reliability was assessed using Cronbach’s alpha, with a threshold value of 0.7 [62]. To evaluate sampling adequacy and model suitability, the Kaiser–Meyer–Olkin (KMO) measure (≥0.7) and Bartlett’s test of sphericity (χ^2^) were applied [62]. Constructs were extracted based on an eigenvalue threshold of 1. Subsequently, descriptive statistics, including means and standard deviations (SD), were calculated for the study variables. A correlation matrix was also analyzed to examine the relationships among the constructs.

For hypothesis testing, Hayes’s Process Macro Model 7 was employed with ordinary least squares estimation and 5000 bootstrap samples. This estimation method was selected because it does not require the normality assumption, thereby reducing the likelihood of bias in inference [66]. To further investigate the moderating effects of nutrition disclosure and chatbot information, the simple slope method was applied.

## 4. Results

### 4.1. Validity and Reliability of Measurement Items

Table 3 reports the results of the factor analysis. The Kaiser–Meyer–Olkin (KMO) value and the chi-square statistic from Bartlett’s test of sphericity exceeded the recommended thresholds, confirming sampling adequacy and the appropriateness of the factor model. All factor loadings were greater than 0.5, and Cronbach’s alpha values exceeded the threshold of 0.7, indicating satisfactory reliability for all constructs (Hair et al., 2010 [62]). These results suggest that the validity and reliability of the measures were ensured. Table 3 also presents the descriptive statistics of the study variables. The mean and standard deviation (SD) values were as follows: health risk (mean = 2.348, SD = 0.979), fatigue (mean = 2.348, SD = 0.979), loyalty (mean = 1.995, SD = 0.987), nutrition disclosure (mean = 3.956, SD = 1.063), and chatbot information (mean = 3.223, SD = 0.996).

### 4.2. Correlation Matrix and Results of Hypotheses Testing

Table 4 presents the correlation matrix of the study variables. Loyalty was significantly correlated with fatigue (r = −0.420, *p* < 0.05), health risk (r = −0.319, *p* < 0.05), nutrition disclosure (r = 0.179, *p* < 0.05), and chatbot information (r = 0.319, *p* < 0.05). Fatigue was positively correlated with health risk (r = 0.397, *p* < 0.05). In addition, chatbot information was positively correlated with nutrition disclosure (r = 0.228, *p* < 0.05). These results provide preliminary support for the hypothesized relationships. Specifically, the negative correlations of loyalty with fatigue and health risk align with H2 and H3, while the positive correlation between fatigue and health risk is consistent with H1. Furthermore, the positive associations of loyalty with nutrition disclosure and chatbot information suggest potential moderating roles for these variables, which were further examined through hypothesis testing using Hayes’s PROCESS Macro Model 7.

Table 5 presents the results of the hypotheses testing. All three models were statistically significant based on F-values (*p* < 0.05). Loyalty was significantly influenced by health risk (β = −0.195, *p* < 0.05) and fatigue (β = −0.375, *p* < 0.05). In addition, health risk exerted a significant positive effect on fatigue (β = −0.175, *p* < 0.05). The interaction terms also showed significant effects. Specifically, Health risk × Nutrition disclosure (β = 0.129, *p* < 0.05) and Health risk × Chatbot information (β = 0.102, *p* < 0.05) significantly affected fatigue, confirming the moderating roles of nutrition disclosure and chatbot information. Taken together, these findings indicate that all hypotheses were supported, with the exception of H1.

**Figure 2:**
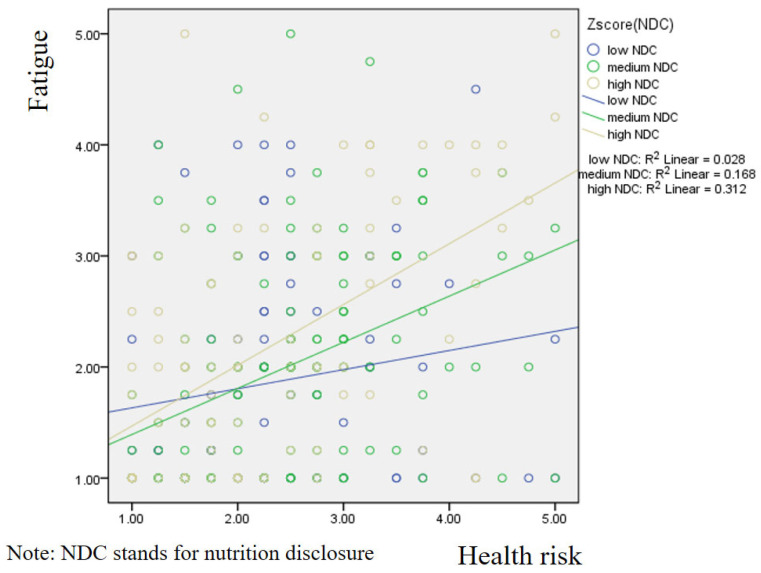
Simple slope method focusing on the moderating role of nutrition disclosure.

Figure 2 presents the results of the simple slope analysis, which illustrates the moderating role of nutrition information disclosure. The findings indicate that consumers with higher perceptions of nutrition information disclosure exhibited greater sensitivity to the effect of health risk on fatigue. In other words, when nutritional information was perceived as being more transparently disclosed, the positive relationship between health risk and fatigue became stronger.

**Figure 3:**
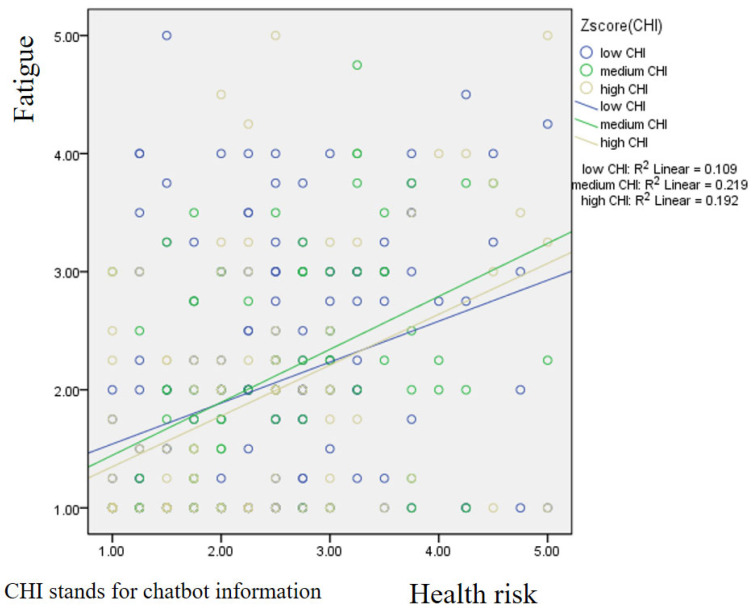
Simple slope method focusing on the moderating role of chatbot information.

Figure 3 displays the results of the simple slope analysis for the moderating role of chatbot information. The findings reveal that consumers with higher perceptions of chatbot information exhibited the steepest positive effect of health risk on fatigue, whereas those with lower perceptions of chatbot information demonstrated a more gradual slope. This suggests that the availability and use of chatbot-provided information amplify the extent to which perceived health risk translates into consumer fatigue.

## 5. Discussion

The purpose of this research was to examine the effects of health risk on fatigue and loyalty among food delivery application users. The results indicated that consumers exhibited a relatively low perception of health risk (mean = 2.348). Their perceived level of fatigue related to using food delivery applications was also relatively low (mean = 1.995). In contrast, consumer loyalty was relatively high. These findings suggest that consumers tend to perceive the foods offered through food delivery applications as posing limited health risks and that they experience only minor fatigue from app usage. One possible explanation for this outcome is the demographic composition of the sample, as a large proportion of respondents were in their forties or younger, and younger consumers may perceive fewer risks or be less susceptible to fatigue in digital environments.

The hypothesis testing results further revealed that perceptions of health risk negatively influenced loyalty but did not have a significant effect on fatigue. In other words, while concerns about the health risks of food may discourage continued use of delivery applications, such concerns do not necessarily induce fatigue during the information search process. Moreover, the findings confirmed that fatigue significantly reduces loyalty. This implies that when information search and decision-making require substantial time and effort, consumers may become less inclined to maintain their engagement with food delivery applications.

Next, the moderating effects of nutrition information disclosure and chatbot information were examined. Consumers displayed a somewhat skeptical response toward nutrition information disclosure (mean = 2.63). This outcome may be explained by the fact that, when using food delivery applications, consumers are already processing multiple other types of information—such as photos, reviews, and promotional content—which may lead them to pay relatively less attention to nutritional details. By contrast, the analysis indicated a moderate level of awareness of chatbot information (mean = 3.223).

Lee et al. [67] conducted a study on food delivery apps that provided insights into consumer behavior across various factors, but the results focused primarily on the main effects. Similarly, Ahn [6] conducted an empirical study on food delivery apps, yet did not provide detailed analyses examining moderating effects or other nuanced relationships. With regard to moderating effects, nutrition information disclosure was found to amplify consumer fatigue. Specifically, when consumers perceived food as posing health risks, the simultaneous consideration of nutritional information created a reinforcing effect that heightened fatigue. These findings implied that when perceptions of food as a health threat become more concrete through the disclosure of nutritional information, individuals may experience greater conflict in their decision-making, which in turn could lead to higher consumption of mental energy during the process. A similar pattern emerged for chatbot information: when chatbot-provided information was considered alongside health risk perceptions, consumers experienced greater fatigue. An alternative explanation was that factors such as limited trust in data sources, insufficient personalization, or multi-channel communication overload through chatbots might have contributed to higher fatigue in decision-making, particularly when the food was perceived as unhealthy. This interpretation could be tentatively drawn from the findings.

Taken together, these findings indicate that both nutrition information disclosure and chatbot information can intensify fatigue during the information search process, as consumers are required to process multiple dimensions of information simultaneously.

## 6. Conclusions

Food & Wine [68] reported that Uber Eats incorporated fresh food into its delivery app marketing as part of efforts to promote sustainable development. Such initiatives could be seen as a strategy to address a limitation of food delivery apps—the predominance of less healthy food—and to improve their market reputation. However, empirical evidence on how these efforts affected consumer behavior remained limited. To address this gap, this research examined the effects of food-related health risks within digital food platforms. The study contributed by investigating the interrelationships among health risks, fatigue, loyalty, nutritional information disclosure, and chatbot-provided information in the context of food delivery app usage. Specifically, it assessed the linear associations between health risks, fatigue, and loyalty, thereby providing insights into consumer behavior in technology-mediated food services. In addition, the moderating roles of nutritional information disclosure and chatbot information on the relationship between health risk and fatigue offered further theoretical understanding of how information environments affected consumer responses. Overall, these findings contributed to discussions on consumer fatigue and offered perspectives on its antecedents and dynamics in digital consumption settings.

From a managerial perspective, several implications emerge. Food delivery app operators might be able to allocate resources toward alleviating consumers’ health-related concerns, for example, by offering healthier menu options that mitigate perceived risks associated with food choices. In addition, strategies to reduce consumer fatigue could be essential. Voice-enabled ordering systems, along with thoughtful design of visual layouts and information functions, may help lower the cognitive burden placed on consumers. Furthermore, the provision of nutritional and chatbot-based information requires careful calibration. Excessive or fragmented information might be able to exacerbate fatigue during information processing. Thus, consolidating relevant content within chatbot systems and streamlining the presentation of nutritional details might be able to enhance usability and reduce consumer strain. In addition, developers of digital food platforms should focus their efforts on designing interfaces that present essential information required for consumption in a way that minimizes user effort. Furthermore, appropriate use of artificial intelligence systems could enable the provision of personalized information or stepwise information based on consumer needs, thereby helping to reduce user fatigue.

## 7. Limitations and Suggestions for Future Research

Despite these contributions, several limitations warrant consideration. First, the data were collected exclusively through survey questionnaires, which restricts methodological diversity. Future research should therefore adopt complementary approaches, such as experimental or longitudinal designs, to validate and extend the present findings. Second, the empirical analysis focused solely on U.S. consumers. Given cross-national variations in food culture, technology adoption, and service infrastructure, future studies would benefit from incorporating comparative samples across different countries to enhance external validity and generalizability. Furthermore, the sample exhibited a skewed demographic profile, with over 70% female participants and a predominance of individuals in their 40s. This imbalance was explicitly acknowledged as a limitation, as it may have affected the generalizability of the findings. Future research could thus validate the findings of this work using a larger variety of cases.

## Figures and Tables

**Table 1:** Survey participants’ profile (*N* = 364).

Characteristics	Frequency	Percentage
Male	107	29.4
Female	257	70.6
20s	57	15.7
30s	131	36
40s	137	37.6
50s	34	9.3
>60 years	5	1.4
Monthly household income		
<$2500	100	27.5
$2500–4999	117	32.1
$5000–7499	61	16.8
$7500–9999	28	7.7
≥$10,000	58	15.9
Terminal academic degree		
Less than college	157	43.1
Bachelor’s degree	145	39.8
Graduate degree	62	17
Food delivery app weekly usage frequency		
<1 time	166	45.6
1–2 times	160	44
3–5 times	30	8.2
≥5 times	8	2.2

**Table 2:** Measurement items description.

Attributes	Codes	Measurement Items
Health risk	HER1	Food delivery apps are harmful to health.
	HER2	Food delivery apps are a cause of obesity.
	HER3	Food delivery apps hinder health promotion.
	HER4	Food delivery apps provide unhealthy food.
Fatigue	FAT1	Using food delivery apps is mentally tiring.
	FAT2	Using food delivery apps consumes a lot of mental energy.
	FAT3	Using food delivery apps is mentally demanding.
	FAT4	Using food delivery apps causes mental fatigue.
Loyalty	LOY1	I will continue to use food delivery apps.
	LOY2	I intend to keep using food delivery apps.
	LOY3	I am willing to continuously use food delivery apps.
	LOY4	I have the intention to continue using food delivery apps.
Nutrition disclosure	NDC1	Food delivery apps provide nutritional information about food.
	NDC2	Food delivery apps provide calorie information.
	NDC3	Food delivery apps provide information about ingredients.
	NDC4	Food delivery apps provide health-related information.
Chatbot information	CHI1	The chatbot consultation of food delivery apps is appropriate.
	CHI2	I think the chatbot consultation of food delivery apps is helpful.
	CHI3	The chatbot consultation of food delivery apps is suitable for problem-solving.
	CHI4	The chatbot consultation of food delivery apps provides appropriate information.

**Table 3:** Validity and reliability of the measurement items.

Construct	Code	Loading	Mean (SD)	Cronbach’s α	Eigenvalue	Explained Variance
Health risk	HER1 HER2 HER3 HER4	0.800 0.849 0.869 0.705	2.348 (0.979)	0.848	1.627	8.135
Fatigue	FAT1 FAT2 FAT3 FAT4	0.851 0.888 0.890 0.868	1.995 (0.987)	0.934	3.996	19.981
Loyalty	LOY1 LOY2 LOY3 LOY4	0.902 0.892 0.865 0.913	3.956 (1.063)	0.958	6.081	30.404
Nutrition disclosure	NDC1 NDC2 NDC3 NDC4	0.837 0.889 0.884 0.856	2.863 (0.950)	0.900	1.892	9.459
Chatbot information	CHI1 CHI2 CHI3 CHI4	0.827 0.919 0.877 0.873	3.223 (0.966)	0.913	2.396	2.396

Note: SD stands for standard deviation, the unit of explained variance is percent, total variance explained: 79.959, Kaiser–Meyer–Olkin Measure (KMO) of Sampling Adequacy: 0.854, Bartlett’s Test of Sphericity Approx. Chi-Square: 5912.043 (*p* < 0.01).

**Table 4:** Correlation matrix.

	1	2	3	4	5
**1. Loyalty**	1				
**2. Fatigue**	−0.420 *	1			
**3. Health risk**	−0.319 *	0.397 *	1		
**4. Nutrition disclosure**	0.179 *	0.099	−0.008	1	
**5. Chatbot information**	0.319 *	−0.059	−0.061	0.228 *	1

Note: * *p* < 0.05.

**Table 5:** Results of hypothesis testing.

	Model1 β-Value (t-Value) Fatigue	Model2 β-Value (t-Value) Fatigue	Model3 β-Value (t-Value) Loyalty
Constant	1.607 (4.41) *	1.930 (4.47) *	5.165 (36.33) *
Health risk	0.009 (0.06)	0.059 (0.33)	−0.195 (−3.52) *
Nutrition disclosure	−0.175 (−1.54)		
Chatbot information		−0.261 (−2.11) *	
Health risk × Nutrition disclosure	0.129 (2.74) *		
Health risk × Chatbot information		0.102 (1.99) *	
Fatigue			−0.375 (−6.81) *
F-value	27.25 *	24.24 *	46.30 *
R^2^	0.1851	0.1681	0.2042
Conditional effect of focal predictor	Nutrition disclosure 2.00: 0.268 (3.93) * 3.00: 0.398 (8.29) * 3.75: 0.495 (8.40) *	Chatbot information 2.00: 0.264 (3.19) * 3.00: 0.366 (7.17) * 4.00: 0.469 (7.80) *	
Interaction effect: F-value	7.55 *	3.99 *	

Note: * *p* < 0.05.

## Data Availability

The original contributions presented in this study are included in the article. Further inquiries can be directed to the corresponding author.
